# Processing Validation Metrics of Syva Enzyme-Multiplied Immunoassay Technique (EMIT) Methotrexate Assay for Beckman Coulter System

**DOI:** 10.7759/cureus.34025

**Published:** 2023-01-20

**Authors:** Saurav Nayak, Suprava Patel, Rachita Nanda, Seema Shah, Eli Mohapatra

**Affiliations:** 1 Biochemistry, All India Institute of Medical Sciences, Bhubaneswar, IND; 2 Biochemistry, All India Institute of Medical Sciences, Raipur, IND

**Keywords:** validation, linearity, imprecision, drug monitoring, accuracy

## Abstract

Background: High-dose methotrexate (HDMTX), defined as a dose greater than 500 mg/m^2^, is used to treat a variety of cancers; and though safe, it can cause major toxicity. Syva enzyme-multiplied immunoassay technique (EMIT) methotrexate (MTX) assay (Gurgaon, India: Siemens Healthcare Diagnostics Ltd.) uses a homogeneous enzyme immunoassay method. Low-end precision performances are very important for laboratory methods, especially when their results have clinical significance at these levels.

Methodology: A total of 25 replicates (five replicates per run, for five runs) were analyzed for profiling. Precision, accuracy, linearity, limit of blank, limit of detection, and limit of quantification were determined using existing guidelines. Imprecision profile and limit of quantitation (LoQ) at 10% were determined by fitting data with hyperbolic regression.

Results: The coefficient of variation percentage (CV%) for low, mid, and high-level internal quality control (IQC) was 1.25%, 3.45%, and 1.55%, respectively. Similarly, estimated bias was -4.58%, -3.54%, -7.21% for each level. The assay linearity was maintained from a range of 0.041-1.993 mmol/L with an R^2^ of 0.959. The limit of detection was estimated to be 0.07 mmol/L.

Conclusion: Syva EMIT MTX assay can be precisely and accurately used to measure low levels of serum methotrexate at levels lower than claimed by the manufacturer, aiding in the monitoring of toxicity in patients.

## Introduction

Methotrexate (MTX) is an integral part of the treatment for acute lymphoblastic leukemia (ALL) and is effective against numerous types of cancer, which justifies its inclusion on the World Health Organization's list of essential medicines [1]. Methotrexate is an antimetabolite that inhibits folic acid metabolism. It binds to dihydrofolate reductase (DHFR) and inhibits the conversion of dihydrofolate to tetrahydrofolate by competitive inhibition. Tetrahydrofolate is required for the biosynthesis of thymidine and purines, which are necessary for DNA synthesis. Methotrexate's inhibition of tetrahydrofolate synthesis renders cells incapable of multiplying and producing proteins. Methotrexate is administered at doses that range from 12 mg intrathecally and 20 mg/m^2^ orally, intramuscularly, or intravenously as weekly maintenance chemotherapy for ALL to doses as high as 33,000 mg/m^2^ intravenously in cases of osteosarcoma or certain lymphomas [2]. High-dose methotrexate (HDMTX), which is defined as a dose greater than 500 mg/m^2^, is used to treat a variety of cancers. Although HDMTX may be administered safely to the majority of patients, it can cause severe toxicity [3]. Multiple systemic functions and organs, including neurotoxicity, hepatotoxicity, mucositis, myelosuppression, and nephrotoxicity, are strongly associated with HDMTX exposure. These adverse effects frequently result in the termination or cessation of chemotherapy and increase the risk of relapse [4]. Therefore, depending on the treatment protocol, HDMTX must be administered with rigorously standardized supportive care to avoid toxicity. Thus, accurate measurements of low levels of MTX in cases of prolonged exposure can help prevent any severe morbidity and mortality in patients with delayed methotrexate excretion [3]. Further, to counter MTX toxicity, supplementation of a formulation of reduced folate (leucovorin) is administered to patients and is termed leucovorin rescue [5].

The potential to quantify MTX at concentrations as low as 0.05 mmol/L is crucial for providing an adequate clinical drug monitoring service. Crucial for the successful delivery of leucovorin, a rescue drug administered following MTX therapy is the measurement of low MTX levels. Leucovorin therapy is continued until MTX levels fall below 0.05 mmol/L. In addition, the detection of low MTX concentrations allows for the assessment of toxic concentrations that may exist 72 hours after high-dose delivery of the drug [6]. As enzyme-multiplied immunoassay technique (EMIT) assays have been of importance for therapeutic drug monitoring, it has been long used for the determination of both endogenous and exogenous analytes in such clinical settings [7]. Syva EMIT MTX (Gurgaon, India: Siemens Healthcare Diagnostics Ltd.) assay uses a homogeneous enzyme immunoassay method. It relies on the competitive binding between the exogenous MTX and the drug labeled with glucose-6-phosphate dehydrogenase for the antibody binding site. The Syva EMIT MTX assay utilizes a five-point logit calibration. The range of calibrator values is between 0.2 and 2.0 mmol/L. The analytical range for the Syva EMIT MTX assay is 0.3-2 mmol/L. However, in clinical practice, the levels of MTX are frequently maintained below this limit for leucovorin rescue and need to be measured [8]. Low-end precision performances are very important for laboratory methods, especially when their results have clinical significance at these levels [9].

Hence, in this study, we have tried to establish the validation profile of Syva EMIT MTX assay on Beckman Coulter AU680 auto-analyzer system (Pasadena, CA: Beckman Coulter Inc.) using established methodologies and laboratory practices for proper monitoring and reporting of low values of MTX in serum of patients following HDMTX administration.

## Materials and methods

The quality parameters for MTX assay were studied on a Beckman AU680 autoanalyzer over a period of five days. The Syva EMIT assay provided calibrators were used to calibrate the test assay for the first time. Bio-Rad Lypochek Therapeutic Drug Monitoring (TDM) levels 1 and 2 were used as internal quality control (IQC) material. As the IQC data for the Beckman Coulter AU system was not established, the Bio-Rad unity worldwide report for therapeutic drug monitoring (April 2022) was used. Specifically, for levels 1 and 2 IQC, the data obtained for Syva EMIT based on Roche Cobas 6000/8000/c 311 (Basel, Switzerland: Roche Holding AG) was used. For level 3 IQC, as there was no equivalent data, the EIA Method Group cumulative mean, SD, and CV were used.

Verification of precision

Clinical Laboratory and Standards Institute (CLSI) guidelines EP15A3 were used for precision verification of the Syva EMIT MTX assay [10]. A total of 25 replicates (five replicates per run, for five runs) were analyzed for precision profiling. Level 1, level 2, and level 3 Bio-Rad QC materials were used. Level 3 QC was run in dilution as its mean value is higher than the analytical range of the assay. The verification of analytical performance of measurands as published by Chakravarthy et al. using CLSI EP15A3 guidelines for the process were reproduced [11]. Grubb's test was used to remove any outliers from the replicates [9].

Verification of trueness

Trueness was estimated by estimating the grand mean elicited from the runs and comparison against the peer group data obtained from Bio-Rad unity worldwide report for therapeutic drug monitoring (April 2022). Bias, total allowable error, and sigma metrics were also evaluated in accordance with established methods [12,13].

Linearity

CLSI EP06 guidelines were used to measure the linearity of the Syva EMIT MTX assay [14]. Eight levels of measurement ranging from 0 to 2 mmol/L were used for available concentrations and prepared by dilution and mixing if not available for the required concentration. The final concentrations used were 0 mmol/L (using provided MTX buffer), 0.05 mmol/L (10x dilution of 0.5 mmol/L calibrator), 0.1 mmol/L (2x dilution of 0.2 mmol/L calibrator), 0.2 mmol/L (calibrator), 0.69 mmol/L (0.5× dilution of Bio-Rad Lypochek TDM level 1 QC + 0.5× 1 mmol/L calibrator), 1 mmol/L (calibrator), 1.24 mmol/L (0.1× Bio-Rad Lypochek TDM level 3 QC + 0.9× Bio-Rad Lypochek TDM level 1 QC) and 2 mmol/L (calibrator). Each sample was analyzed in five replicates per run for five runs. Generated linear and non-linear regression equations were analyzed for closeness to true value by R^2^ values.

Low-end performance

Clinical Laboratory and Standards Institute (CLSI) published the EP17-A approved guideline - "protocols for determination of limits of detection and limits of quantitation" in 2004 and has defined low-end performances based on limit of blank, limit of detection, and limit of quantification [9]. Limit of blank (LoB) is thereby defined as "the highest value we expect to see in a series of results on samples that contain no analyte," limit of detection (LoD) is "the actual concentration at which an observed test result is very likely to exceed the LoB and may therefore be declared as detected," whereas the limit of quantitation (LoQ) is defined as "the actual concentration at which the analyte is reliably detected and at which the uncertainty of the observed test result is less than or equal to the goal set by the laboratory" [9].

The methodology as outlined by Armbruster et al. was utilized for estimation of LoB and LoD [15]. Five samples (two assay buffer samples, two patient samples who haven't been administered MTX, and one distilled water sample) were run for five days (LoB = Mean_blank_ + 1.645 × SD_blank_). For replicating, a low serum MTX concentration, just above the LoB, was attained by diluting a 0.2 mmol/L calibrator and running five replicates per run for five runs (LoD = LoB + 1.645 × SD_low concentration sample_) [16]. Five sample concentrations from LoD to approximately two times LoD were run in five replicates for five runs. Imprecision profile and LoQ 10% were determined by fitting data with hyperbolic regression. A 95% confidence interval (95% CI) was evaluated by the inverse regression method after linearization of the hyperbolic function [17]. All data analysis was done using statistical and mathematical formulas incorporated in MS Excel 365 and by using Statistical Package for Social Science (SPSS version 26.0; Chicago, IL, IBM Corp.).

## Results

Table 1 summarizes the result of Grubbs' test with no significant outliers. Thus, no data removal was done, and all the replicates were analyzed. The comparison of imprecision estimates against manufacturer’s claim is represented in Table 2.

**Table 1:** Grubbs' test for outlier detection in replicates. G: Grubb’s test coefficient, which is required to remove outliers

Replicate level	N	Mean	SD	Range (min-max)	Grubbs' critical factor	G for replicates	Acceptable range	Significant outliers
L1	25	0.396	0.006	0.380-0.410	2.663	2.432	0.379-0.412	No
L2	25	1.072	00.34	0.996-1.119	2.663	2.177	0.982-1.162	No
L3	25	7.948	0.108	7.765-8.207	2.663	2.350	7.662-8.235	No

**Table 2:** Comparison of imprecision estimates against manufacturer’s claim. *Manufacturer’s claim, unity worldwide report. **Manufacturer’s claim. IQC: internal quality control, CV% R: within run coefficient of variation percentage, CV% WL: within lab coefficient of variation percentage

IQC	Mean±SD	N	CV% R	Claimed R*	CV% WL	Estimated WL**
Level 1	0.415±0.015	25	2.53	6.8	1.52	8.8, 3.5
Level 2	1.13±0.085	25	1.87	6.8	3.45	8.8, 7.5
Level 3	8.57±0.739	25	1.38	6.8	1.55	8.8, 8.6

The manufacturer obtained CV% for within-run is 6.8% and for between-run is 5.6%. The Unity Worldwide Report provides a CV% of 3.5%, 7.5%, and 8.6% for levels 1, 2, and 3 IQC, respectively. Our precision profiling estimated a CV% of 1.5%, 3.5%, and 1.6% for within-lab precision of levels 1, 2, and 3 IQC, respectively. This is significantly lower compared to the available data.

The levels of IQC had a bias of -4.6%, -3.5%, and -7.2% respectively. All replicates had a total error (TE) less than the accepted total allowable error for methotrexate of 10% [18]. The sigma value of the IQC was also estimated and all the matrices have been tabulated in Table 3. The relative comparison of allowable errors for precision and trueness was estimated. Precision estimates for levels 1 and 3 IQC were optimal but for level 2 were desirable (Table 4). Total allowable error was optimal, desirable, and minimal for levels 1, 2, and 3 IQC, respectively (Table 5).

**Table 3:** Bias profile of replicates. IQC: internal quality control, CV% WL: within lab coefficient of variation percentage, TE: total error

IQC	Target mean	Observed mean	CV% WL	Bias%	TE%	Sigma
Level 1	0.415	0.396	1.52	-4.58	7.09	3.57
Level 2	1.13	1.09	3.45	-3.54	9.23	1.9
Level 3	8.57	7.95	1.55	-7.21	9.77	1.8

**Table 4:** Comparison of CV% and bias% to imprecision and bias factors. IQC: internal quality control, CV% WL: within lab coefficient of variation percentage, L1: level 1, L2: level 2, L3: level 3

IQC	Manufacturer CV% WL	CV% WL	Imprecision factors			Bias%	Bias factors		
			Optimal	Desirable	Minimal		Optimal	Desirable	Minimal
			0.25	0.5	0.75		0.125	0.25	0.375
L1	8.81	1.52	2.20	4.40	6.61	4.58	1.10	2.20	3.30
L2		3.45				3.54			
L3		1.55				7.21			

**Table 5:** Comparison of total error allowable. TEa: total error allowable

Internal quality control	Estimated TEa	Total error allowable		
		Optimal	Desirable	Minimal
Level 1	7.09	4.73	9.47	14.20
Level 2	9.23			
Level 3	9.77			

**Figure 1:**
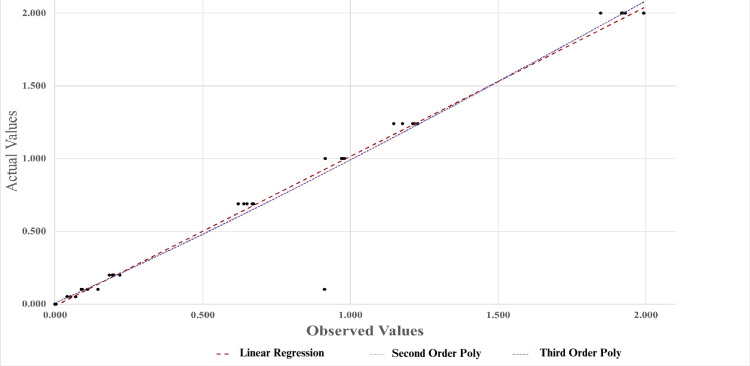
Linear and non-linear estimates for linearity.

The linearity of the assay was estimated by both linear and non-linear regressions. The range of linearity was from 0.041 mmol/L to 1.993 mmol/L. Non-linear polynomial regressions of the 2nd and 3rd orders were used. R^2^ values 0.959, 0.960, and 0.960 were estimated for linear, 2nd order polynomial, and 3rd order polynomial, respectively. The linearity data has been represented in Table 6 and Figure 1.

**Table 6:** Summary of linear and non-linear regression analysis. Linear regression equation: y = (1.0291x) - 0.0137 2nd order polynomial regression equation: y = (0.0562x^2^) + (0.9294x) + 0.0047 3rd order polynomial regression equation: y = (-0.0202x^3^) + (0.1147x^2^) + (0.8894x) + 0.0076

Model		y-Intercept	x	x^2^	x^3^	R^2^
Linear	Coefficient	-0.014	1.029	NA	NA	0.959
2nd order polynomial	Coefficient	0.005	0.929	NA	NA	0.960
3rd order polynomial	Coefficient	-0.020	0.115	0.889	0.008	0.960

**Figure 2:**
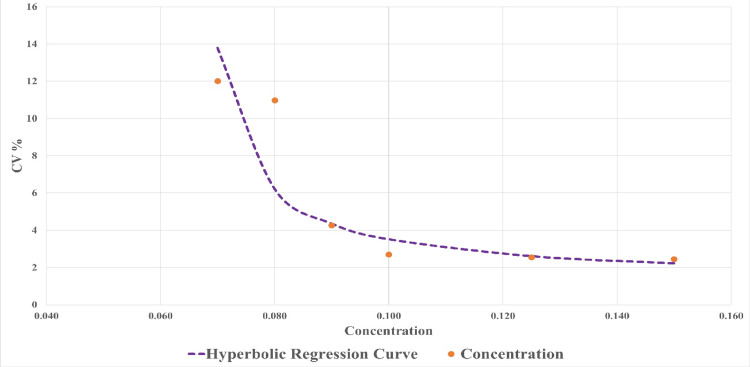
Hyperbolic regression curve for determination of LoQ. LoQ: limit of quantitation

**Figure 3:**
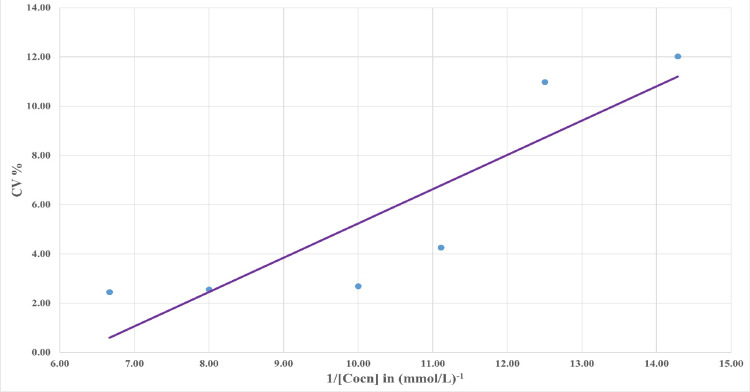
Linearized imprecision profile for determination of LoQ with 95% CI. LoQ: limit of quantitation

The limit of blank was estimated to be 0.03 mmol/L. The limit of detection was estimated to be 0.07 mmol/L. The limit of quantification was estimated from the hyperbolic regression curve to be 0.073 mmol/L and 0.085 mmol/L for cut-off CV% of 10% and 5%, respectively (Figure 2). Linearization of the hyperbolic curve provided a 95% CI of 0.012 to 0.319 for LoQ at a 10% CV cut-off (Figure 3).

## Discussion

The quality of a patient's result is inversely proportional to the laboratory error rate. Periodically, a laboratory should review the quality of its errors in order to determine the extent of their impact on patient safety [11]. Therefore, the measurement of precision and trueness of a test procedure is of paramount importance. HDMTX toxicity may be fatal for the patient, and hence an apt measurement of MTX levels is warranted. Beckman Coulter AU systems are one of the generally used automated laboratory systems; hence validation of Syva EMIT MTX assay was highly required.

Our coefficient of variation for levels 1 and 2 IQC was 1.52% and 3.45%, respectively. The values are significantly less than the manufacturers' and Bio-Rad unity worldwide report's provided values. Our precision was comparable to that of Chavan et al. where levels 1 and 2 IQC were 0.60 and 2.8%, respectively [8]. It is, however, much lesser compared to the CV% range of 0.88-9.43 reported by Borgman et al. [6] and 5.31-8.43 reported by Suh-Lailam et al. [19]. Another study by Lim et al. also had a higher CV% than our study (1.8-11.2%) [20]. In the same study, the between-run CV% was 8.1%, 1.3%, and 3.5% for low, medium, and high-level controls, respectively, similar to our study.

Shi et al. studied the Syva EMIT MTX assay in Siemens Viva-E autoanalyzer and had a bias ranging from -1.60% to -5.67% [21]. Our study, however, had a higher range of bias ranging from -3.54% to -7.21%. The high bias is comparable to the reports of Borgman et al. who had biases as high as 21.9% and 9.46% for medium-level control materials [6].

The manufacturer claims a lower linearity level of 0.3 mmol/L. However, our study estimated linearity from 0.07 to 1.99 mmol/L, which is more sensitive than that reported by Chavan et al. (0.17-1.98 mmol/L) [8]. Lim et al. reported a lower linearity limit of 0.6 mmol/L ranging up to 1.92 mmol/L with R^2^=0.955, similar to our study [20]. The estimated LoD in all cases was lower than the manufacturers' claimed 0.3 mmol/L. Our study estimated LoD of 0.07 mmo/L, while Wilson et al. estimated 0.20 mmol/L, Chavan et al. had 0.17 mmol/L, and Lim et al. estimated 0.06 mmol/L [8,22,20]. Thus, our assay sensitivity was corroborated by previous studies.

Limit of quantification is highly important because it decides what lowest value of an analyte can be measured with a high degree of performance, especially when lower concentrations matter from a clinical perspective. There is a lack of proper LoQ validation profiles for Syva EMIT MTX assay in previous literature, hence our study takes primary importance in this regard. We estimated an LoQ of 0.073 mmol/L and 0.850 mmol/L for cut-off of 10% and 5% CV, respectively. Linearized curve analysis provided a lower limit of 0.012 mmol/L (R^2^=0.778), comparable to the lower limits estimated by Lim et al. [20].

As methotrexate measurement by Syva EMIT assay did not have data for IQC and worldwide values for the Beckman AU system, for evaluation of verification matrices, we had to rely on rather generic data as provided by the manufacturer and unity report. This was one of the limitations of our study.

## Conclusions

Verification of performance metrics of methods elevates in importance when the analyte in question is of toxic proportions, even at a lower serum concentration. Hence, evaluating analytical performance through the estimation of precision, trueness, linearity, and low-level performance will help in aiding the clinical implications of methotrexate treatment aptly with minimal error. It will also help to set a benchmark for other laboratories using other instruments for processing methotrexate, as well as be a reproduction of the importance of CLSI guidelines and other relevant statistical examinations to provide proper and verified metrics to all exploring laboratories to meet their needs for their measured biochemical tests.
